# Sodium-glucose cotransporter 2 inhibitor dapagliflozin prevents ejection fraction reduction, reduces myocardial and renal NF-κB expression and systemic pro-inflammatory biomarkers in models of short-term doxorubicin cardiotoxicity

**DOI:** 10.3389/fcvm.2024.1289663

**Published:** 2024-05-16

**Authors:** V. Quagliariello, M. L. Canale, I. Bisceglia, M. Iovine, A. Paccone, C. Maurea, M. Scherillo, A. Merola, V. Giordano, G. Palma, A. Luciano, F. Bruzzese, F. Zito Marino, M. Montella, R. Franco, M. Berretta, D. Gabrielli, G. Gallucci, N. Maurea

**Affiliations:** ^1^ Division of Cardiology, Istituto Nazionale Tumori—IRCCS—Fondazione G. Pascale, Napoli, Italia; ^2^ Cardiology Division, Azienda USL Toscana Nord-Ovest, Versilia Hospital, Lido di Camaiore, Italy; ^3^ Integrated Cardiology Services, Department of Cardio-Thoracic-Vascular, Azienda Ospedaliera San Camillo Forlanini, Rome, Italy; ^4^ ASL NA1, UOC Neurology and Stroke Unit, Ospedale del Mare, Naples, Italy; ^5^ Cardiology Department, San Pio Hospital, Benevento, Italy; ^6^ Department of Pharmacy, University of Salerno, Salerno, Italy; ^7^ SSD Sperimentazione Animale, Istituto Nazionale Tumori—IRCCS—Fondazione G. Pascale, Napoli, Italy; ^8^ Pathology Unit, University of Campania “Luigi Vanvitelli”, Naples, Italy; ^9^ Department of Clinical and Experimental Medicine, University of Messina, Messina, Italy; ^10^ U.O.C. Cardiologia, Dipartimento Cardio-Toraco-Vascolare, Azienda Ospedaliera San Camillo Forlani-ni, Roma—Fondazione per il Tuo Cuore—Heart Care Foundation, Firenze, Italy; ^11^ Cardio-Oncology Unit, Istituto di Ricovero e Cura a Carattere Scientifico (IRCCS), Referral Cancer Center of Basilicata, Rionero in Vulture, Italy

**Keywords:** cancer, dapagliflozin, cardio-oncology, cardioprotection, doxorubicin, NF-kB

## Abstract

**Background:**

Anthracycline-mediated adverse cardiovascular events are among the leading causes of morbidity and mortality in patients with cancer. Sodium-glucose cotransporter 2 inhibitors (SGLT2i) exert multiple cardiometabolic benefits in patients with/without type 2 diabetes, chronic kidney disease, and heart failure with reduced and preserved ejection fraction. We hypothesized that the SGLT2i dapagliflozin administered before and during doxorubicin (DOXO) therapy could prevent cardiac dysfunction and reduce pro-inflammatory pathways in preclinical models.

**Methods:**

Cardiomyocytes were exposed to DOXO alone or combined with dapagliflozin (DAPA) at 10 and 100 nM for 24 h; cell viability, iATP, and Ca^++^ were quantified; lipid peroxidation products (malondialdehyde and 4-hydroxy 2-hexenal), NLRP3, MyD88, and cytokines were also analyzed through selective colorimetric and enzyme-linked immunosorbent assay (ELISA) methods. Female C57Bl/6 mice were treated for 10 days with a saline solution or DOXO (2.17 mg/kg), DAPA (10 mg/kg), or DOXO combined with DAPA. Systemic levels of ferroptosis-related biomarkers, galectin-3, high-sensitivity C-reactive protein (hs-CRP), and pro-inflammatory chemokines (IL-1*α*, IL-1β, IL-2, IL-4, IL-6, IL-10, IL-12, IL17-α, IL-18, IFN-γ, TNF-α, G-CSF, and GM-CSF) were quantified. After treatments, immunohistochemical staining of myocardial and renal p65/NF-kB was performed.

**Results:**

DAPA exerts cytoprotective, antioxidant, and anti-inflammatory properties in human cardiomyocytes exposed to DOXO by reducing iATP and iCa^++^ levels, lipid peroxidation, NLRP-3, and MyD88 expression. Pro-inflammatory intracellular cytokines were also reduced. In preclinical models, DAPA prevented the reduction of radial and longitudinal strain and ejection fraction after 10 days of treatment with DOXO. A reduced myocardial expression of NLRP-3 and MyD-88 was seen in the DOXO-DAPA group compared to DOXO mice. Systemic levels of IL-1β, IL-6, TNF-α, G-CSF, and GM-CSF were significantly reduced after treatment with DAPA. Serum levels of galectine-3 and hs-CRP were strongly enhanced in the DOXO group; on the other hand, their expression was reduced in the DAPA-DOXO group. Troponin-T, B-type natriuretic peptide (BNP), and N-Terminal Pro-BNP (NT-pro-BNP) were strongly reduced in the DOXO-DAPA group, revealing cardioprotective properties of SGLT2i. Mice treated with DOXO and DAPA exhibited reduced myocardial and renal NF-kB expression.

**Conclusion:**

The overall picture of the study encourages the use of DAPA in the primary prevention of cardiomyopathies induced by anthracyclines in patients with cancer.

## 1 Introduction

Anthracyclines are associated with dose-dependent cardiotoxicity (1). Cancer patients treated with anthracyclines at 400 and 700 mg/m^2^ are exposed to a 5% and 48% risk of congestive heart failure, respectively (2). Mechanisms of acute and chronic anthracycline-mediated adverse events involve ferroptosis, endothelial damages, apoptosis, fibrosis, and myocardial inflammation mediated by overexpression of NF-kB mediated pathways (3, 4). Notably, short-term-induced myocardial damages of doxorubicin (DOXO) are well reported in clinical scenarios, resulting in the need for cardioprotective strategies in primary prevention in patients with cancer (5). A wide spectrum of cardioprotective drugs is proposed, including sacubitril/valsartan, beta blockers, and nutraceuticals; however, no effective risk reductions were seen in these patients (6).

Sodium-glucose cotransporter type 2 inhibitors (SGLT2i) have beneficial properties, including the improvement of systolic and diastolic functions (7), increase in calcium homeostasis, reduction of afterload and oxidative stress, improvement of mitochondrial functions in cardiomyocytes, and increase in ketone bodies, resulting in improved energy metabolism of cardiac cells, reduction of insulin and uric acid levels as well as of epicardial and visceral fat (8, 9). The most studied SGLT2is are empagliflozin (EMPA), dapagliflozin (DAPA), canagliflozin (CANA), and ertugliflozin (ERTU), which differ in their SGLT2 binding avidity, resulting in different clinical outcomes (10).

DAPA is a selective SGLT2i with multiple beneficial properties in patients with cardiovascular diseases (CVD) (10). In the DECLARE-TIMI trial, DAPA reduced cardiovascular death and hospitalization for heart failure in patients with type 2 diabetes mellitus (T2DM) (11). In the DAPA-HF TRIAL, DAPA reduced heart failure and death from cardiovascular causes in patients with heart failure and reduced ejection fraction in patients with and without T2DM (12). In the DEFINE-trial, DAPA improved heart failure-related health status and reduced natriuretic peptides in patients with heart failure with reduced ejection fraction (13). In the DELIVER trial, in patients with heart failure and preserved ejection fraction, DAPA significantly reduced cardiovascular death and urgent heart failure visits in patients with T2DM (14). A very recent trial of cancer patients with T2DM treated with anthracyclines and gliflozins reduced heart failure admissions, new cardiomyopathies, arrhythmias, and heat failure incidence (15).

The aim of the present study was to test, for the first time, whether DAPA could affect the myocardial and renal NF-κB expression, systemic levels of 12 cytokines, growth factors, troponin, and B-type natriuretic peptide NT-pro-BNP in preclinical models of short-term doxorubicin cardiotoxicity, preventing ejection fraction reduction.

## 2 Materials and methods

### 2.1 Cardioprotective properties of DAPA in human cardiomyocytes

To evaluate the cytoprotective effects of DAPA in human cardiomyocytes (AC16 adult human cells; Sigma Aldrich, Milan, Italy), mitochondrial dehydrogenase activity was quantified through a modified MTT [3-(4,5-dimethyldiazol-2-yl)-2,5-diphenyl tetrazolium bromide] method, known as MTS assay, according to the manufacturer's instructions (Dojindo Molecular Technologies Inc., Rockville, MD, USA). Briefly, AC16 cells were cultured in Dulbecco's Modified Eagle's Medium/Nutrient Mixture F-12 Ham (DME/F-12) supplemented with 10% fetal bovine serum (FBS; 10,000 cells/well) at 37°C in a humidified 5% CO_2_ atmosphere. After 24 h of appropriate growth, cells were unexposed (control) or exposed to DOXO (range 0.1–50 µM) or DAPA (10 or 100 nM) or both in combination for 24 h, in line with the literature (16). Notably, cellular DAPA doses were chosen according to the literature (close to the plasma levels of DAPA after oral administration in adults) (17–20). After treatment, cells were then washed three times with phosphate buffered solution (PBS) at pH 7.4 and then incubated with 100 μl of an MTT solution (0.5 mg/ml in cell culture medium) for 4 h at 37°C. Absorbance readings were acquired at a wavelength of 450 nm with the Tecan Infinite M200 plate-reader (Tecan Life Sciences Home, Männedorf, Switzerland) using I-control software (Tecan). Relative cell viability (%) was calculated with the following formula = [A]test/[A]control × 100, where “[A]test” is the absorbance of the test sample, and “[A]control” is the absorbance of the control cells incubated solely in culture medium (21).

### 2.2 Intracellular Ca^++^ levels and ATP levels

DOXO-mediated cardiovascular injuries involve high intracellular calcium levels induced by intracellular Reactive Oxygen Species (iROS) (22). Intracellular Ca^2+^ in AC16 cells was quantified through the fluorescence dye Fluo-3 AM, according to the manufacturer's protocol. Cardiomyocytes were untreated (control) or treated with DOXO at 0.5 µM alone or combined with DAPA (10 or 100 nM) for 12 h. Notably, the DOXO concentration used in these experiments (0.5 μM) was chosen since the plasma concentration of anthracyclines in cancer patients has been reported to fluctuate in the range of 0.3–1 μM during infusion (23–25). After incubation, the cells were loaded with 5 µM Fluo-3 AM at 37°C for 30 min in the dark, and then washed three times with PBS (pH 7.4) to remove the excess dye. Fluo-3 chelated with Ca^++^ induces fluorescence detected by a spectrofluorometer (excitation/emission wavelengths 488 and 525 nm, respectively). Instead, intracellular adenosine-5'-triphosphate (ATP) levels were quantified through ENLITEN® ATP Assay System (Promega Italia S.r.l, Milan, Italy) according to the literature (26). Briefly, cardiomyocytes were untreated (control) or treated for 24 h, as described previously; after treatments, 100 μl of lysis/assay solution provided by the manufacturer was added to confluent cell cultures in 96-well plates. After the plates were shaken for 1 min and incubated for 10 min at 23°C, luminescence was measured in a microplate luminometer (Thermo Fisher, Milan, Italy). Data were expressed as relative units (r.u.) according to the literature (27).

### 2.3 Lipid peroxidation products (MDA and 4-HNA)

Anthracyclines exert cardiotoxic effects through the induction of ferroptosis, a cell death induced by lipid peroxidation (28). AC16 cells were grown as described above; subsequently, 5,000 cells/well were seeded in a 24-well plate and allowed to grow for 24 h and exposed to DOXO (0.5 µM) or DAPA (10 or 100 nM). After centrifugation at 800 × g for 5 min, malondialdehyde (MDA) and 4-hydroxy 2-hexenal (4-HNA) were quantified though commercial kits with a spectrophotometer according to the manufacturer's protocols (Sigma Aldrich, Milan, Italy).

### 2.4 NLRP-3 and MyD-88 expression

Cardiomyocytes were treated as described in Section 2.2; after treatment, the cells were harvested and lysed in complete lyses buffer (50 mM Tris–HCl, pH 7.4, 1 mM EDTA, 100 mM NaCl, 20 mM NaF, 3 mM Na3 VO4, 1 mM PMSF, and protease inhibitor cocktail). After centrifugation, supernatants were collected and treated to the quantification of MyD88 [Human MyD88 ELISA Kit (ab171341); Abcam, Milan, Italy] and NLRP3 [Human NLRP3 ELISA Kit (OKEH03368); Aviva Systems Biology, San Diego, CA, USA]. For the human MyD88 ELISA, the sensitivity was <10 pg/ml and the range of detection was 156–10,000 pg/ml; for the human NLRP3 ELISA assay, the sensitivity was <0.078 ng/ml and the range of detection was 0.156–10 ng/ml (29).

### 2.5 Intracellular pro-inflammatory cytokines assay

The expression of pro-inflammatory cytokines, such as IL-6, IL-8, and IL-1β, was performed through enzyme-linked immunosorbent assay (ELISA) methods, in line with the literature (30). Briefly, AC16 cells were treated as described in Section 2.2 for 12 h; after treatment, the cells were lysed as described in Section 2.4 and quantification of IL-1β, IL-6, and IL-8 was performed through selective ELISA kits according to the manufacturer's instructions (Sigma Aldrich, Milan, Italy).

### 2.6 Morphological changes and mitochondrial activity of cardiomyocytes exposed to anthracyclines and DAPA through a Confocal Laser Scanning Microscope

Morphological changes and mitochondrial activity of human cardiac cells were studied through a Confocal Laser Scanning Microscope (EZ-C1-Nikon). Briefly, human cardiac cells were untreated (control) or treated with DOXO alone or combined with DAPA for 24 h. After incubation, cardiomyocytes were fixed in 4% formaldehyde (10 min) and then incubated in 1% BSA/10% normal goat serum/0.3 M glycine in 0.1% PBS-Tween20 for 1 h to permeabilize the cells and block non-specific protein–protein interactions. The cardiomyocytes were then incubated with an anti-Mitochondria antibody (113-1)—BSA and Azide free (Abcam ab92824, Milan, Italy) 5 µg/ml overnight at ±4°C. As a secondary antibody (green), a DyLight® 488 goat anti-mouse IgG (H ± L) (ab96879, Abcam, Milan, Italy) was used at a dilution of 1/250 for 1 h. Membrane staining was obtained using Concanavalin A Tetramethylrhodamine Conjugate (Invitrogen, Life Technology, Milan, Italy) at a final concentration of 100 µg/ml. Through a confocal microscope (C1-Nikon) equipped with EZ-C1 software for data acquisition and 60× oil immersion objective, intracellular mitochondria were imaged through excitation/emission at 488/518 nm and cell membrane through excitation/emission at 555/580 nm (31).

### 2.7 Preclinical model of short-term doxorubicin cardiotoxicity

In total, 24 female C57Bl/6 mice (aged 6–7 weeks) were purchased from ENVIGO, San Pietro al Natisone (Italy). The mice were housed six per cage and maintained on a 12-h light/dark cycle (lights on at 7.00 a.m.) in a temperature-controlled room (22°C ± 2°C) and with food and water *ad libitum*. Preclinical experimental protocols were in accordance with EU Directive 2010/63/EU for animal experiments, and Italian D.L.vo 26/2014 law, were approved by the Ministry of Health (authorization number 1,467/17-PR of the 13-02-2017) and the institutional ethics committees: by Organismo preposto al benessere degli animali (OPBA). After 1 week of growth, the mice were randomized for weight-adjusted treatment. The mice were divided into four experimental groups (*n* = 6/group): (i) 100 μl saline solution (Saline); (ii) DOXO at 2.17 mg/kg/day through intraperitoneal administration (i.p.); (iii) DAPA 10 mg/kg/day through oral gavage; and (iv) DOXO/DAPA in combination (at the same concentration of each drug tested alone). Treatments were performed according to recently published studies with the aim of assessing the cardioprotective effects of ranolazine (32) and empagliflozin (33) against DOXO-induced cardiotoxicity for 10 days (34). Low doses of anthracyclines in preclinical models were used in line with other cardioprotective outcome studies (35, 36). Moreover, this is a short-term doxorubicin treatment study that is able to detect echocardiographic changes and systemic and myocardial inflammation (33, 37) due to acute pro-inflammatory and cardiotoxicity phenomena induced by DOXO, in line with other studies by Tocchetti et al. (38) and similar studies of preclinical models of cardiotoxicity (39–41). The chosen dose of DAPA (10 mg/kg/day through oral gavage) was assessed according to several preclinical studies available in the literature (18, 42–45) as well as other preclinical studies with other SGLT2is in cardio-oncology, such as empagliflozin (34).

### 2.8 Transthoracic echocardiography and blood analysis

A non-invasive transthoracic echocardiography through a Vevo 2,100 high-resolution imaging system (40-MHz transducer; Visualsonics, Toronto, ON, Canada) was performed in line with the literature (32, 34, 46). The mice were anesthetized with tiletamine (0.09 mg/g), zolazepam (0.09 mg/g), and 0.01% atropine (0.04 ml/g). Later, the animals were sedated and placed in a supine position on a temperature-controller surgical table to maintain a rectal temperature of 37°C and continual ECG monitoring was obtained via limb electrodes. Cardiac function was evaluated at basal conditions and at 2 and 10 days of treatment. Left ventricular echocardiography was assessed in parasternal long-axis views at a frame rate of 233 Hz. Notably, we measured the strain in parasternal views because the apical view is difficult to perform in small animals (39); this method was in line with other studies for speck tracking echocardiography (STE) analyses that were performed on parasternal long-axis B-mode loops using a VisualSonics Vevo 2100 system (VisualSonics) (47, 48). Image depth, width, and gain settings were optimized to improve image quality. End-systole and end-diastole dimensions were defined as the phases corresponding to the ECG T wave, and to the R wave, respectively. M-mode LV internal dimensions, diastolic (LVID,d) and LV internal dimensions, and systolic (LVID,s) dimensions were averaged from 3–5 beats. LVID,d and LVID,s were measured from the LV M-mode at the mid-papillary muscle level. Fractional shortening percentage (% FS) was calculated as [(LVID,d − LVID,s)/LVID,d] × 100, and ejection fraction percentage (% EF) was calculated as [(EDvol − ESvol)/EDvol] × 100. The strain was expressed as percentage. The analysis started with acquired B-mode loops and were imported into the Vevo Strain software. Three consecutive cardiac cycles were selected, and the endocardium traced. Upon adequate tracing of the endocardium, an epicardial trace was added. The ST-based strain allowed for the assessment of strains specific to six myocardial segments per LV view. Internally, 10 or more points were measured for each of the six segments, resulting in a total of 48 data points. Strain and strain rate (SR) are useful in the detection of regional myocardial function. The strain was also evaluated on long-axis views as radial and longitudinal. Radial strain (RS), defined as the percent change in myocardial wall thickness, is a positive curve reflecting increasing myocardial thickness during systole and diminishing wall thickness during diastole and represents myocardial deformation toward the center of the LV cavity. Longitudinal strain (LS) detects the percent change in the length of the ventricle, typically measured from the endocardial wall in the long-axis view. The myocardial deformation rate, expressed in 1/s, was also calculated. Importantly, during echocardiography, the heart rate of the mice was carefully monitored and was similar among all experimental groups, i.e., approximately 500 bmp (range 490–510 bmp), according to the literature (49). Echocardiographic analyses were performed following the “Small Animal Echocardiography using the Vevo® 2100 Imaging System” guidelines as well as other previous studies in models of preclinical cardio-oncology (34, 50–52). The mice analyzed through echocardiography after 10 days of treatment with DOXO to measure left ventricular systolic function, heart rate, and cardiac output were previously described (34, 38, 46) and in accordance with the recommendations of the American Society of Echocardiography (53). Blood glucose determination was performed by puncture of the tail vein before and after treatments using a glucometer (Model NC).

### 2.9 Myocardial NLRP-3 and MyD-88 expression

After treatment, the hearts were fully weighed. Subsequently, a left ventricular sample was cut, fixed, and embedded in paraffin for histological studies (on left ventricular histological effects, as described in Section 2.6). The remaining heart tissue was homogenized and lysed for quantitative analyses of NLRP-3 and Myd-88. In detail, the tissue was snap-frozen in dry ice until tissue homogenization was performed in a proper lysis buffer (0.1 M PBS, pH 7.4 + 1% Triton X-100 + protease inhibitor cocktail) and processed using a high-intensity ultrasonic liquid processor (54, 55). The homogenates were centrifuged at 4°C and supernatants were used for the NLRP-3 and Myd-88 analyses through NLRP3 (Mouse NLRP3 ELISA Kit, OKEH05486; Aviva Systems Biology) and MyD88 (Mouse MyD88 ELISA Kit, OKEH03397; Aviva Systems Biology).

#### 2.9.1 Systemic troponin-T, BNP, NT-Pro-BNP, galectin-3, and high-sensitivity C-reactive protein levels

At the end of the treatment, blood sampling via cardiac puncture was performed to quantify the biomarkers of cardiotoxicity [Troponin-T, BNP, N-Terminal Pro-Brain Natriuretic Peptide (NT-Pro-BNP)] and the biomarkers of systemic inflammation [galectin-3 and high-sensitivity C-reactive protein (hs-CRP)]. Briefly, mouse troponin-T, BNP, and NT-Pro-BNP were quantified through the Mouse Troponin-T, cardiac muscle (TNNT2) ELISA kit (CusaBio, Houston, TX, USA), Mouse BNP ELISA Kit (A77763, Antibodies Stockholm, Sweden), and Mouse NT-Pro-BNP ELISA Kit (Abbexa, Cambridge, UK). Galectin-3 was quantified through the Galectin 3 Mouse ELISA Kit (Thermo Scientific, Milan, Italy) and hs-CRP was determined through the Mouse hs-CRP ELISA Kit (Elabscience Biotechnology Co., USA) (55).

#### 2.9.2 Systemic levels of ferroptosis biomarkers and cytokines

At the end of the treatment, blood sampling via cardiac puncture was performed to quantify two biomarkers of ferroptosis, products of lipid peroxidation, MDA, and 4-HNA using commercial kits with a spectrophotometer according to the manufacturer's protocols (39) [MAK085, Sigma Aldrich, Milan, Italy, for MDA; Lipid Peroxidation (4-HNE) Assay Kit, ab238538, AbCam, Italy]. In total, 12 cytokines and growth factors (IL-1α, IL-1β, IL-2, IL-4, IL-6, IL-10, IL-12, IL17-α, IFN-γ, TNF-α, G-CSF, GM-CSF) were quantified through a mouse cytokine Multiplex Assay kit (pg/ml; Qiagen, USA) (56).

#### 2.9.3 IHC staining of NF-kB in left ventricular heart samples and kidney tissue

Left ventricular heart samples and kidney tissue were fixed in 4% paraformaldehyde for 1 h and then kept at 4°C until paraffin embedding. Cardiac and kidney paraffin sections (with a thickness of 4 μm) were hydrated, microwaved for 8–15 min in 10 mM sodium citrate (pH 6.0) for antigen retrieval, and then probed with a rabbit antibody against murine p65/NF-kB (1:100, ab16502; Ab Cam, Milan, Italy). Immunolabeled sections were then incubated with goat anti-rabbit second antibody conjugated to horseradish peroxidase and treated with the EnVision + diaminobenzidine kit (DAB; Dako, Glostrup, Denmark) using standard protocols (57). The stained sections were analyzed by two independent observers, at least five different areas for each specimen were evaluated, and the mean was assessed. NF-kB IHC was categorized as positive or negative, as well as an overall proportion of cells (10%) with positive nuclear staining in the studied field at a magnification ×100. IHC scoring was based on the nuclear staining intensity according to the literature as follows: score 0, no nuclear staining; score 1, weak staining; score 2, moderate staining; and score 3, strong staining (58–60).

#### 2.9.4 Statistical analyses

Continuous data were expressed as mean ± SD. Non-parametric tests were used both for paired and unpaired comparisons. A repeated measures ANOVA was used for all baseline to end-of-study comparisons. A *p*-value <0.05 was considered significant.

## 3 Results

### 3.1 Cytoprotective and anti-inflammatory effects of DAPA in cardiomyocytes exposed to anthracyclines

**Figure 1:**
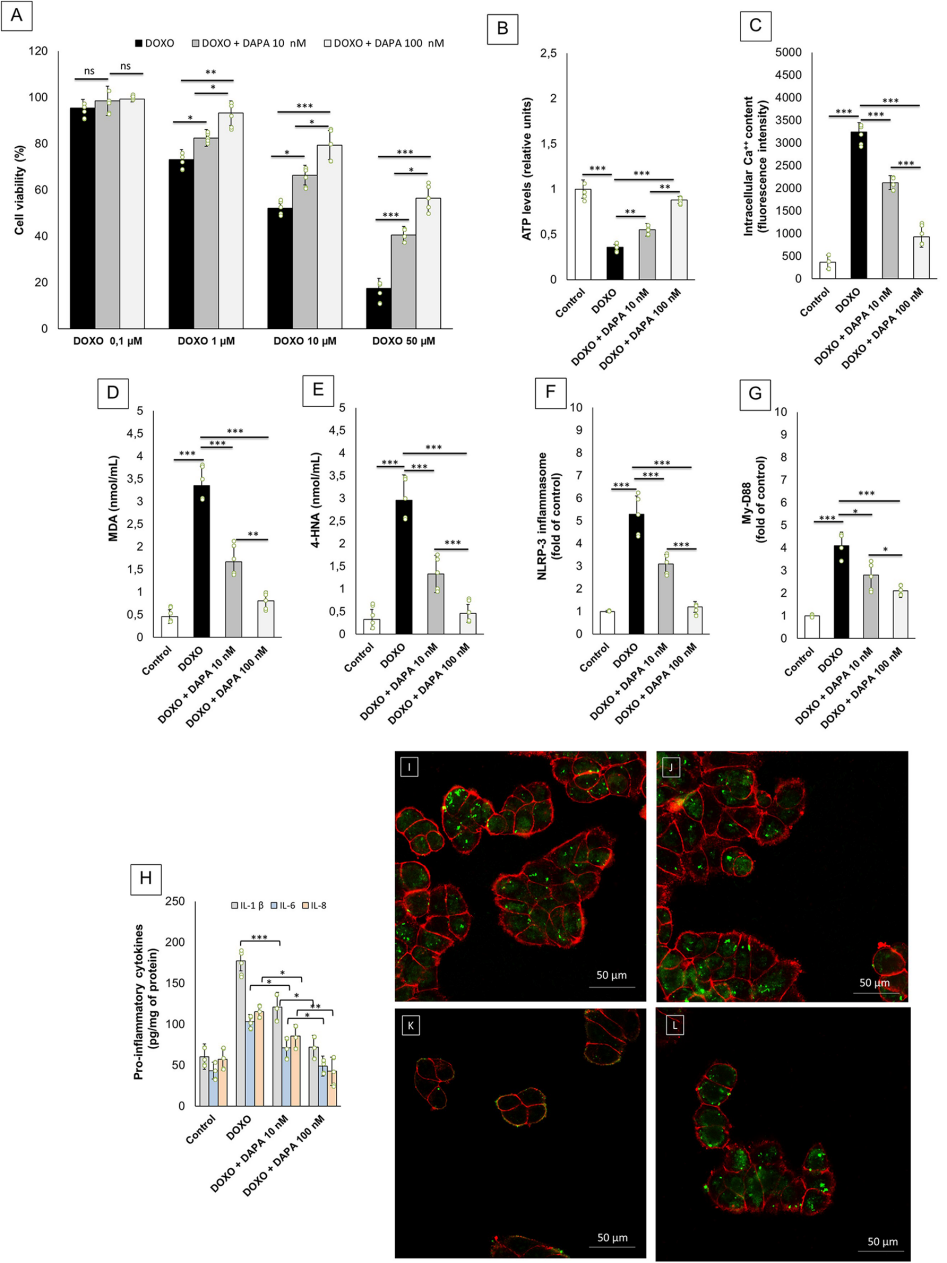
DAPA exerts cardioprotective properties in human cardiomyocytes exposed to DOXO. (**A**) Cell viability (% of control) of cardiomyocytes exposed to DOXO (0.1, 1, and 10 µM) alone or combined to DAPA (10 or 100 nM) for 24 h. ATP levels (**B**) (relative units), intracellular Ca^++^ content (**C**) (fluorescence intensity), MDA (**D**) and 4-HNA (**E**) (nmol/ml), NLRP-3 (**F**) and MyD-88 (**G**) (fold of control) in human cardiomyocytes unexposed (control) or exposed for 24 h to DOXO (0.5 µM) alone or combined to DAPA (10 or 100 nM). Pro-inflammatory cytokines (**H**) (IL-1, IL6, and IL-8, pg of cytokine/mg of protein) in human cardiomyocytes unexposed (control) or exposed for 24 h to DOXO (0.5 µM) alone or combined to DAPA (10 or 100 nM). One-way ANOVA. Values are expressed as ±SD. ***P < 0.001; **p < 0.01; *p < 0.05; ns: not significant. Confocal scanning laser microscope (**I–L**) of human cardiomyocytes unexposed (**I**) or exposed to DAPA (**J**) or DOXO (**K**) or DOXO-DAPA (**L**) for 24 h. Green signals: mitochondrial staining; Red signals: cell membrane. Scale bar: 50 μM.

As described in the literature, anthracyclines exert cardiotoxic effects through lipid peroxidation, high intracellular Ca^++^ levels, mitochondrial damage, and myocardial inflammation mediated by NLRP-3/MyD-88/cytokine pathways (61). In line with the literature, DAPA showed cytoprotective properties in cardiomyocytes exposed to DOXO for 24 h (Figure 1A), increasing significantly their cell viability [i.e., of 20% and 38% for DAPA 10 and 100 nM, respectively, compared to only DOXO (50 µM) treated cells; *p* < 0.001]. Cardiac cells exposed to DOXO drastically reduced intracellular ATP levels compared to untreated cells (−64% vs. control; *p* < 0.001) (Figure 1B); instead, DAPA increased their content by 11% and 52% compared to DOXO groups (*p* < 0.001 for both). Intracellular Ca^++^ were significantly increased in cardiac cells exposed to DOXO (3,244.4 ± 203.3 vs. 367.6 ± 153.8 a.u.; *p* < 0.001) (Figure 1C); co-incubation with DAPA at 10 and 100 nM drastically reduced iCa^++^ levels compared to DOXO (2,123.5 ± 155.5 and 927.8 ± 234.4 vs. 3,244.4 ± 203.3 a.u., respectively; *p *< 0.001). Lipid peroxidation products MDA and 4-HNA (Figures 1D,E) were significantly increased in cardiomyocytes exposed to DOXO (3.35–2.96 nmol/ml vs. 0.5 nmol/ml; *p* < 0.001); co-incubation with DAPA reduced their intracellular levels in a concentration-dependent manner, demonstrating antioxidant properties (*p* < 0.001 vs. DOXO groups). Intracellular levels of NLRP-3 and Myd-88, were also drastically increased after exposure to DOXO (Figures 1F,G) (∼5.3 and 4.1 times compared to untreated cells; *p* < 0.001 for both). Notably, co-incubation with DAPA significantly reduced their levels (NLRP-3 levels in DAPA 100 nM were comparable to untreated cells; *p* < 0.001), indicating anti-inflammatory effects. Intracellular cytokine levels also changed significantly between groups (Figure 1H); in detail, IL-1β levels in DAPA 10 and 100 nM compared to the DOXO only group were 121.1 ± 17.7 and 72.2 ± 14.4 pg/mg of protein versus 177.3 ± 12.2, respectively (*p* < 0.001 for both); instead, IL-6 levels in DAPA 10 and 100 nM compared to the DOXO only group were 71.1 ± 13.2 and 48.8 ± 11.8 pg/mg of protein versus 103.3 ± 8.6, respectively (*p* < 0.001 for both); IL-8 levels in DAPA 10 and 100 nM, compared to the DOXO only group were 85.5 ± 14.3 and 42.7 ± 17.2 pg/mg of protein versus 115.2 ± 8.3, respectively (*p* < 0.001 for both). These results are in line with other studies on SGLT2i cardioprotective properties and indicate cytoprotective, antioxidant, and anti-inflammatory properties of DAPA in human cardiomyocytes. Confocal images clearly showed morphological changes in human cardiomyocytes exposed to DOXO (Figure 1K), with an initial loss of cell–cell interactions and lower fluorescent signal related to the cell membrane (red signals) compared to untreated cells (Figure 1I), characteristic of cellular atrophy induced by anthracyclines. Furthermore, mitochondrial staining (green signals) was significantly reduced in the DOXO group compared to the DAPA group (Figure 1J), indicating a loss of the number and functionality of mitochondria. Notably, co-incubation with DOXO and DAPA prevents the loss of cardiomyocyte morphology (Figure 1L) and prevents the reduction of mitochondrial staining, showing a high and significant green fluorescence compared to only DOXO exposed cells.

### 3.2 DAPA did not affect serum glucose in non-diabetic mice exposed to anthracyclines

As reported in other studies, gliflozins did not affect serum glucose in non-diabetic mice but were able to reduce oxidative-related products both systemically and in heart tissue (62–64). In brief, DAPA-treated mice had a blood glucose of 187.6 ± 29.3 mg/dl vs. 192.8  ± 37.1 mg/dl in untreated mice (no differences were seen between groups; *p* = 0.71). No differences in blood glucose were seen between the DOXO and DOXO-DAPA groups (213.47 ± 41.3 mg/dl vs. 198.6 ± 32.2 mg/dl, respectively; *p* = 0.43) These results are in line with those of other studies (8, 65) confirming that DAPA did not significantly change blood glucose in non-diabetic mice.

### 3.3 DAPA improves cardiac function in DOXO-induced cardiotoxicity

**Figure 2:**
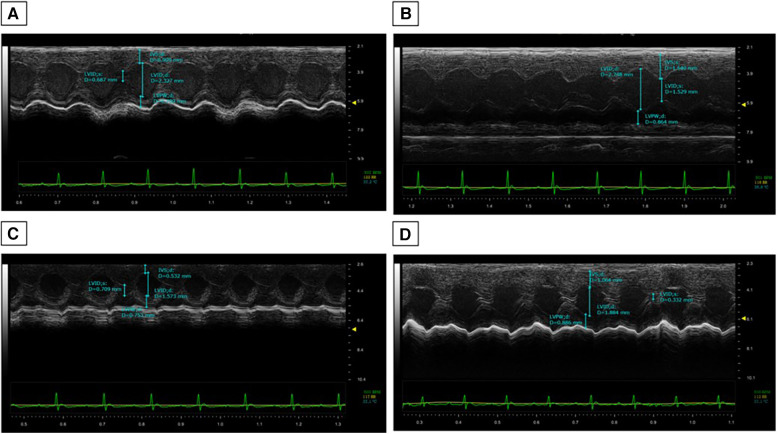
DAPA attenuated DOXO-induced impairment of cardiac systolic function. Echocardiography was performed on all the mice after 10 days of DOXO injection. Representative M-mode of long-axis echocardiographic images for measurements of the IVSd (mm), LVPWd (mm), LVIDd (mm), and LVIDs (mm) of Saline (**A**), DOXO (**B**), DAPA (**C**), and DOXO-DAPA (**D**) mice.

The cardiac function analysis clearly shows the cardiotoxicity of DOXO even after 10 days of treatment (Table 1). Specifically, significant reductions in EF (%), FS (%), radial strain (Pk%), and longitudinal strain (Pk%) were seen compared to the controls (DOXO vs. Saline; *p* < 0.001). In addition, a slight but not significant increase in LV mass was seen (Table 1). Instead, the DAPA group showed preservation of cardiac function compared to the Saline group, confirming the cardiac benefits in preclinical models. On the other hand, the DOXO-DAPA group showed a significant improvement in EF (%), FS (%), radial strain (Pk%), and longitudinal strain (Pk%) versus DOXO (DOXO-DAPA vs. DOXO; *p* < 0.001). The representative M-mode of long-axis echocardiographic images (Figure 2) for measurements of the intraventricular septum thickness in diastole (IVSd) (mm), the thickness of the rear wall of the left ventricle (LVPWd) (mm), LVIDd (mm), and LVIDs (mm) of mice clearly indicates that DAPA (Figure 2D) improves cardiac functions during DOXO therapy compared to the DOXO only group (Figure 2B). Hearts weighed after necropsy showed a slight increase in heart weight in the DOXO groups than the Saline and DOXO groups, probably due to high inflammation and hypertrophy induced by anthracycline therapy. Notably, DAPA did not significantly reduce heart weight compared to DOXO alone.

**Table 1:** Cardiovascular parameters of study groups, such as (saline), DOXO 2.17 mg/kg/day, DAPA 10 mg/kg/day, and DOXO-DAPA in association (*n* = 6 for each group).

Cardiovascular parameters	Saline	DOXO	*p*-value	DAPA	DOXO-DAPA	*p*-value
			Saline vs. DOXO			DOXO vs. DOXO-DAPA
IVS,d-D (mm)	0.61 ± 0.06	0.59 ± 0.04	0.512	0.63 ± 0.05	0.62 ± 0.07	0.383
LVID,d-D (mm)	2.1 ± 0.34	2.5 ± 0.22	0.036	1.9^ ^± 0.25	2.2 ± 0.4	0.138
LVPW,d-D (mm)	0.62 ± 0.09	0.67 ± 0.14	0.478	0.64 ± 0.12	0.63 ± 0.17	0.665
LV Mass (mg)	50,2 ± 3.4	53.5 ± 2.7	0.09	49.4 ± 2.5	52.4 ± 2.1	0.449
LVID,s-D (mm)	1.23 ± 0.1	1.79 ± 0.21	0.0002	1.24 ± 0.4	1.33 ± 0.24	0.005
EF (%)	93.4 ± 1,2	78.5 ± 1.5	<0.0001	94.3^ ^± 2.1	89.7 ± 1.6	<0.0001
FS (%)	63.2 ± 2.1	47.3 ± 1.8	<0.0001	64.2^ ^± 1.5	59.7 ± 1.3	<0.0001
Radial strain (Pk%)	36.3 ± 3.4	14.3 ± 2.6	<0.0001	38.3^ ^± 2.3	32.4 ± 3.1	<0.0001
Longitudinal Strain (Pk%)	−22.4 ± 2.6	−12.6 ± 3.1	0.0001	−26.2^ ^± 5.2	−21.4 ± 2.1	0.0007
Heart weight (g)	0.12 ± 1.4	0.19 ± 2.1	0.947	0.08 ± 1.6	0.13 ± 1.2	0.952

Cardiac function parameters IVS,d-D (mm), LVID,d-D (mm), LVID,s-D (mm), LVPW,d-D (mm), EF (%), FS (%), LV Mass (mg), LV Vol,d (µl), and LV Vol,s (µl) were analyzed through echocardiography (Vevo/2100). Heart weight among four groups after necropsy (g). Two-way ANOVA with a Bonferroni *post-hoc test* was performed for statistical analysis. Values are expressed as mean ±SD. *p*-values are shown for differences between DOXO and Saline as well as DOXO-DAPA vs. DOXO groups.

### 3.4 DAPA reduces NF-kB expression in myocardial and kidney tissue during DOXO therapy

**Figure 3:**
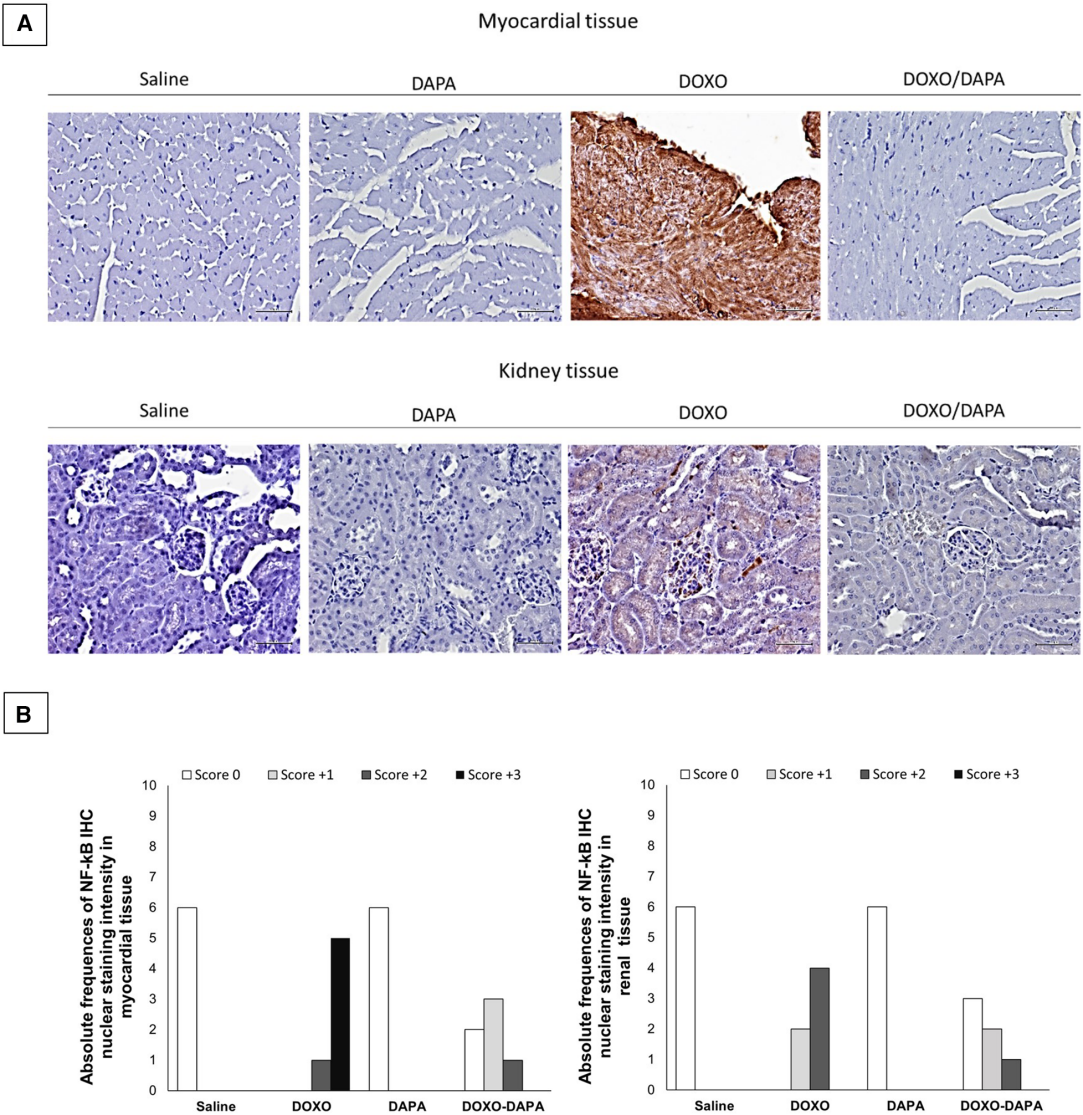
(**A**) Myocardial (up) and kidney (down) p65/NF-kB expression in mice treated with saline solution (saline), DAPA 10 mg/kg/day, DOXO 2.17 mg/kg/day. or DAPA associated to DOXO (*n* = 6 for each group). Scale bar: 5 µm; (**B**) Absolute frequencies of NF-KB IHC nuclear intensity in myocardial and renal tissues of mice treated with saline solution (Saline), DAPA 10 mg/kg/day, DOXO 2.17 mg/kg/day, or DAPA associated to DOXO. NF-kB IHC was categorized as positive or negative, as well as an overall proportion of cells (10%) with positive nuclear staining in the studied field at ×100 magnification. IHC scoring was based on the nuclear staining intensity, as follows: score 0, no nuclear staining; score 1, weak staining; score 2, moderate staining; score 3, strong staining.

Histological analyses were performed to evaluate the anti-inflammatory effects of DAPA in preclinical models of DOXO cardiotoxicity (Figure 3). In line with the literature (4, 65), DOXO induces tissue overexpression of p65/NF-kB. It is very interesting to note that DAPA totally changed the renal and cardiac inflammatory picture, strikingly reducing the expression of p65/NF-kB, preserving the tissue microstructure of cardiomyocytes and kidney (Figure 3). In more detail, from a histological point of view, the administration of DOXO/DAPA did not show morphological alterations detectable with hematoxylin and eosin staining (Figure 3A). Cellular morphology remained essentially unchanged in terms of nucleus/cytoplasm ratio and volume of individual sarcomeres. The likely reason could be attributed to the short duration of anthracycline administration; thus further studies will be needed to assess any morphological changes upon long-term administration (as specified in the Discussion section). Instead, quantitative NF-kB staining indicates a high score (±3) of nuclear NF-kB staining in myocardial tissue in the DOXO group, indicating a pro-inflammatory effect induced by anthracycline therapy (Figure 3B); notably, the DOXO-DAPA group showed a significant reduction of nuclear NF-kB staining score compared to the DOXO group with no high score (±3) and only weak (±1) and moderate (±2) staining seen, confirming DAPA-related myocardial anti-inflammatory properties in these preclinical models (Figure 3B). A renal tissue analysis was performed as an internal control, considering that SGLT2is have mainly been used as antidiabetic drugs acting on the proximal convoluted tubule of the kidney (expressing SGLT-2), where they block the reabsorption of glucose and sodium, favoring the urinary excretion of glucose (7, 8), Subsequent studies have also demonstrated the expression of SGLT2 in cardiac tissues, broadening their clinical spectrum of action in the prevention of cardiovascular diseases (33, 34).

### 3.5 DAPA attenuates systemic inflammation induced by DOXO

**Figure 4:**
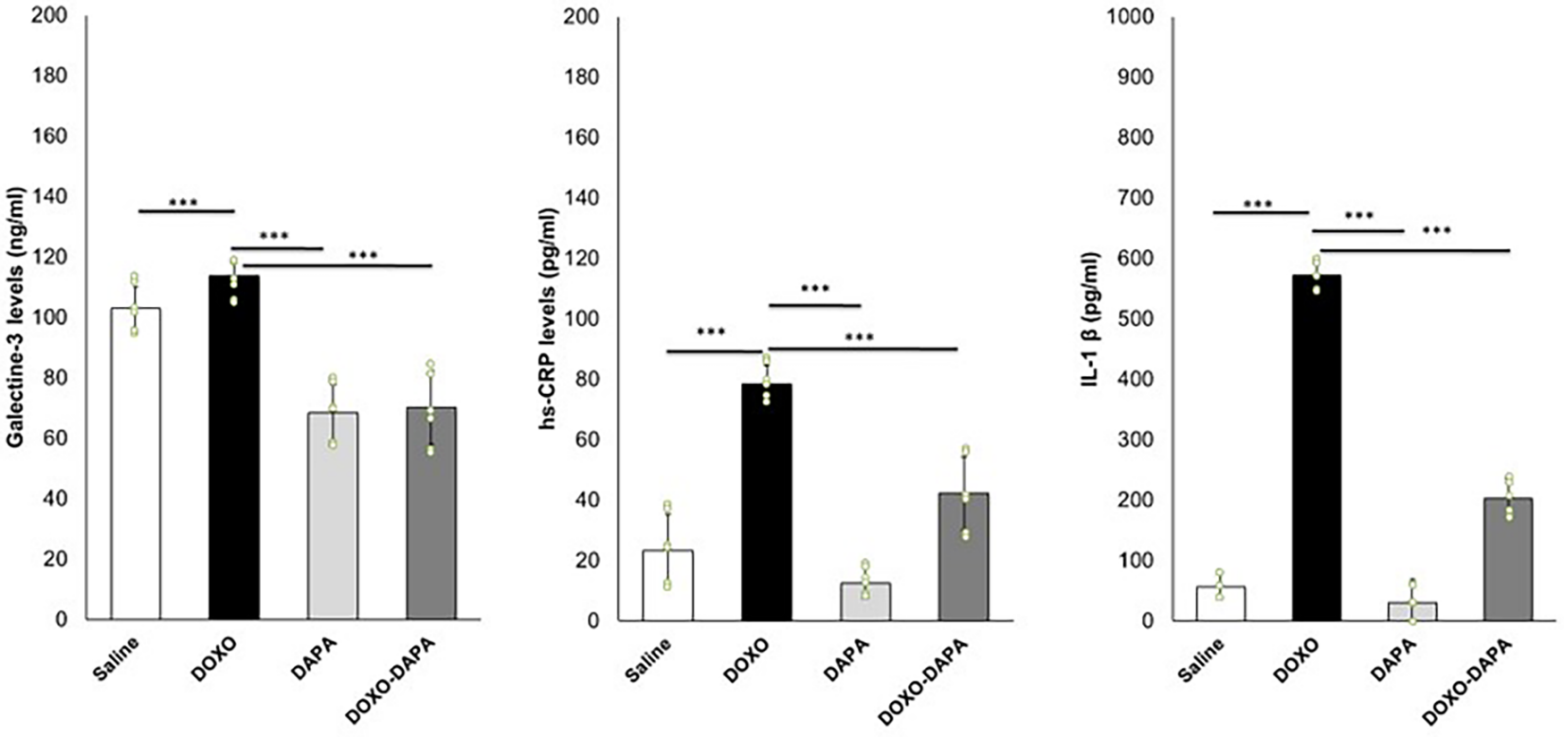
DAPA reduced systemic levels of galectin-3 (ng/ml), hs-CRP (pg/ml), and IL-1 (pg/ml) during treatment with DOXO. Mice were treated with saline solution (control), DOXO 2.17 mg/kg/day, DAPA 10 mg/kg/day, and DOXO-DAPA in association (*n* = 6 for each group). One-way ANOVA. Values are expressed as mean ±SD. ***p < 0.001; **p < 0.01; *p < 0.05; ns, not significant.

DOXO induces systemic inflammation in cancer patients (66, 67). We investigated the systemic anti-inflammatory effects of DAPA during DOXO therapy. In line with literature, DOXO increased serum Galectin-3, IL-1, and hs-CRP levels compared to the Saline group. DAPA is able to reduce hs-CRP, IL-1, and Galectin-3 significantly, indicating systemic anti-inflammatory effects (Figure 4).

### 3.6 DAPA reduces troponin-T, BNP, and NT-pro-BNP during DOXO therapy

In preclinical models, it has been observed that DOXO treatment can lead to an increase in troponin and BNP, Troponin-T, and NT-pro-BNP levels (68–70). In line with the literature, short-term DOXO therapy increased the systemic levels of cardiotoxicity biomarkers compared to saline (Table 2). DAPA treatment did not significantly change the troponin and natriuretic peptide levels compared to saline, confirming no cardiac adverse events. Interestingly, in the DOXO-DAPA group, a significant reduction in Troponin-T (0.21 ± 0.05 vs. 0.46 ± 0.06; *p* < 0.001); BNP (128.6 ± 16.4 vs. 182.3 ± 42.1; *p* < 0.001); and NT-pro-BNP (1,112.7 ± 68.3 vs. 1,432.3 ± 72.1; *p* < 0.001) was seen, showing the cardioprotective properties of DAPA.

**Table 2:** Biomarkers of cardiotoxicity, troponin-T (ng/ml), BNP (pg/ml), NT-pro-BNP (pg/ml) quantified after 10 days of treatment with saline solution (control) DOXO 2.17 mg/kg/day, DAPA 10 mg/kg/day, and DOXO-DAPA in association (*n* = 6 for each group).

Biomarkers	Saline	DOXO	*p*-value	DAPA	DOXO-DAPA	*p*-value
			Saline vs. DOXO			DOXO vs. DOXO-DAPA
Troponin-T (ng/ml)	0.19 ± 0.03	0.46 ± 0.06	<0.001	0.16 ± 0.04	0.21 ± 0.05	<0.001
BNP (pg/ml)	124.6 ± 34	182.3 ± 42.1	0.024	113.4 ± 21.3	128.6 ± 16.4	0.015
NT-pro-BNP (pg/ml)	1,085.4 ± 62.1	1,432.3 ± 72.1	<0.001	1,012.5 ± 57.5	1,112.7 ± 68.3	<0.001

One-way ANOVA; Values are expressed as mean ±SD. *p*-values are shown for differences between Saline and DOXO or DOXO and DOXO-DAP groups.

### 3.7 DAPA attenuates myocardial NLRP3 and MyD-88 expression in DOXO-induced cardiotoxicity

**Figure 5:**
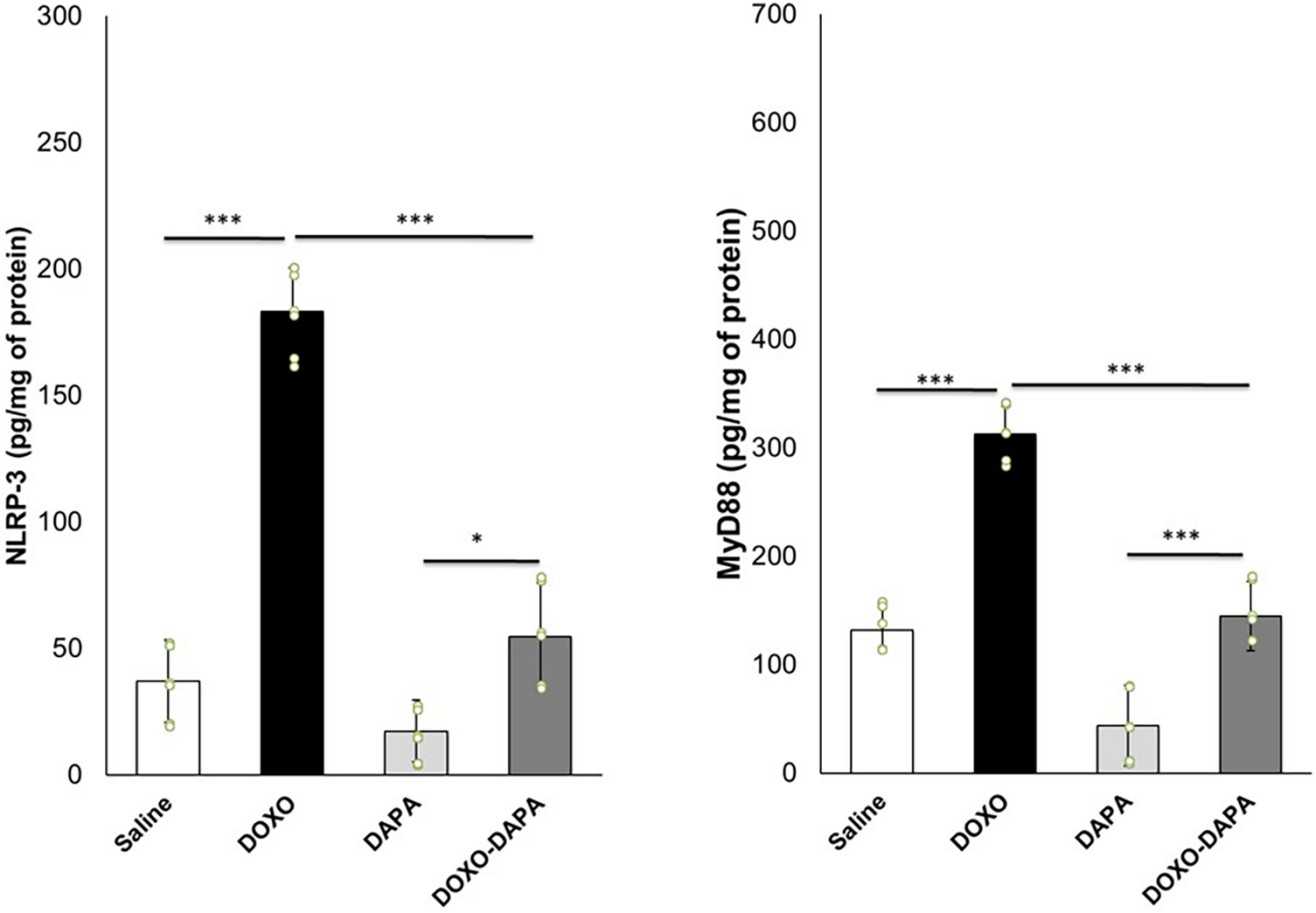
NLRP3 and MyD-88 expression (pg/mg of protein) in myocardial tissues of mice treated with saline solution (control), DOXO 2.17 mg/kg/day, DAPA 10 mg/kg/day, and DOXO-DAPA in association (*n* = 6 for each group). One-way ANOVA. Values are expressed as mean ±SD. ***p < 0.001; **p < 0.01; *p < 0.05; ns, not significant.

In recent years, there has been growing interest in understanding the involvement of NLRP3 inflammasome and MyD-88 activation in various pathological conditions, including cardiotoxicity induced by DOXO (71, 72). In line with the literature, DOXO therapy increased the myocardial levels of NLRP3 and MyD-88 compared to saline (Figure 5). A significant reduction of NLRP3 and MyD-88 were seen in the DOXO-DAPA group versus the DOXO group, demonstrating the anti-inflammatory effects of DAPA during DOXO therapy.

### 3.8 DAPA reduces myocardial ferroptosis and pro-inflammatory cytokines and chemokines in mice exposed to anthracyclines

Emerging evidence suggests that NLRP3 inflammasome activation can induce or contribute to ferroptosis in preclinical models through the induction of cytokines able to damage mitochondria (73, 74). Lipid peroxidation products (MDA and 4-HNA) can serve as markers of ferroptosis (75, 76). During ferroptosis, the peroxidation of polyunsaturated fatty acids (PUFAs) in cellular membranes generates reactive lipid species, such as MDA and 4-HNA (77). As shown in Figure 5, myocardial levels of MDA and 4-HNA were strongly enhanced in the DOXO group compared to saline (*p* < 0.001). DAPA significantly reduced lipid peroxidation without DOXO and combined with DOXO, demonstrating the antioxidant and preventive properties of ferroptosis in myocardial tissues. Moreover, a pro-inflammatory cytokine profile was seen in the DOXO group (Figure 5). Instead, DAPA totally reversed the inflammatory picture induced by DOXO, reducing IL-1, IL6, TNF-a, and IL-17 levels.

## 4 Discussion

Dapagliflozin is a SGLT2i primarily used for the management of type 2 diabetes mellitus (78); however, its use has expanded to the field of cardiology due to its cardiovascular benefits (79). Dapagliflozin has shown efficacy in reducing the risk of cardiovascular events and improving heart failure outcomes (80). Here are some of the key uses of dapagliflozin in cardiology: recent cardiovascular outcomes trials have demonstrated that DAPA can reduce the risk of major adverse cardiovascular events (MACE) in patients with established cardiovascular disease (81). These events include heart attack, stroke, and cardiovascular-related death. Dapagliflozin has been shown to provide cardiovascular protection in high-risk patients, including those with a history of heart disease (82).

Moreover, DAPA is able to reduce heart failure hospitalizations and improves outcomes in patients with heart failure, both with reduced ejection fraction (HFrEF) and preserved ejection fraction (HFpEF) through the reduction of fluid, thus improving cardiac function (83). Notably, DAPA has also demonstrated benefits in preserving kidney function and reducing the risk of kidney disease progression in patients with or without diabetes (83, 84). This can be particularly relevant in patients with cardiovascular disease who may have concomitant renal toxicities. In brief, DAPA helps lower blood glucose levels by inhibiting SGLT2, which reduces glucose reabsorption in the kidneys and increases urinary glucose excretion; therefore, by improving glycemic control, it can have additional indirect benefits on cardiovascular health (85, 86). While DAPA is primarily indicated for the management of diabetes, some recent studies suggests that SGLT2is may exerts anticancer effects and could potentially be used as an adjunct therapy for certain types of tumors, including breast and liver tumors (87). Briefly, one of the proposed mechanisms of action for dapagliflozin in cancer is its ability to reduce glucose availability to cancer cells; considering cancer cells often exhibit increased glucose uptake compared to normal cells (depending on the type and biology of tumors), the inhibition of glucose reabsorption in the kidneys of DAPA could potentially deprive cancer cells of a key energy source (88, 89). In addition, DAPA induces euglycemic diabetic ketoacidosis (DKA), which has been shown to selectively inhibit the growth of some cancer cells, such as triple negative breast cancer and hormone-responsive breast cancer (90, 91). However, only cellular and preclinical studies are available and further research is needed to establish its clinical significance.

Anthracyclines are a class of chemotherapy drugs commonly used in the treatment of various types of cancer, including breast cancer, lymphoma, and leukemia (92). While anthracyclines have shown effectiveness in fighting cancer, they exert significant dose-related cardiotoxicity (93). Recent studies have examined the potential benefits of using SGLT2is in patients who have received anthracycline-based chemotherapy (94). These studies have shown promising results regarding the cardiac outcomes of such patients.

A recent study investigated the effects of EMPA on cardiac function in patients with breast cancer treated with anthracyclines (95). The study found that EMPA improved LVEF and reduced biomarkers of heart failure. In addition, EMPA is able to reduce the incidence of heart failure and cardiovascular death in these patients. Another recent study evaluated the cardioprotective effects of DAPA in patients with breast cancer receiving anthracycline-based chemotherapy (15, 96). Briefly, the authors concluded that DAPA preserved LVEF and reduced markers of cardiac injury compared to placebo. In that case, DAPA was also associated with a lower risk of heart failure and cardiovascular events.

**Figure 6:**
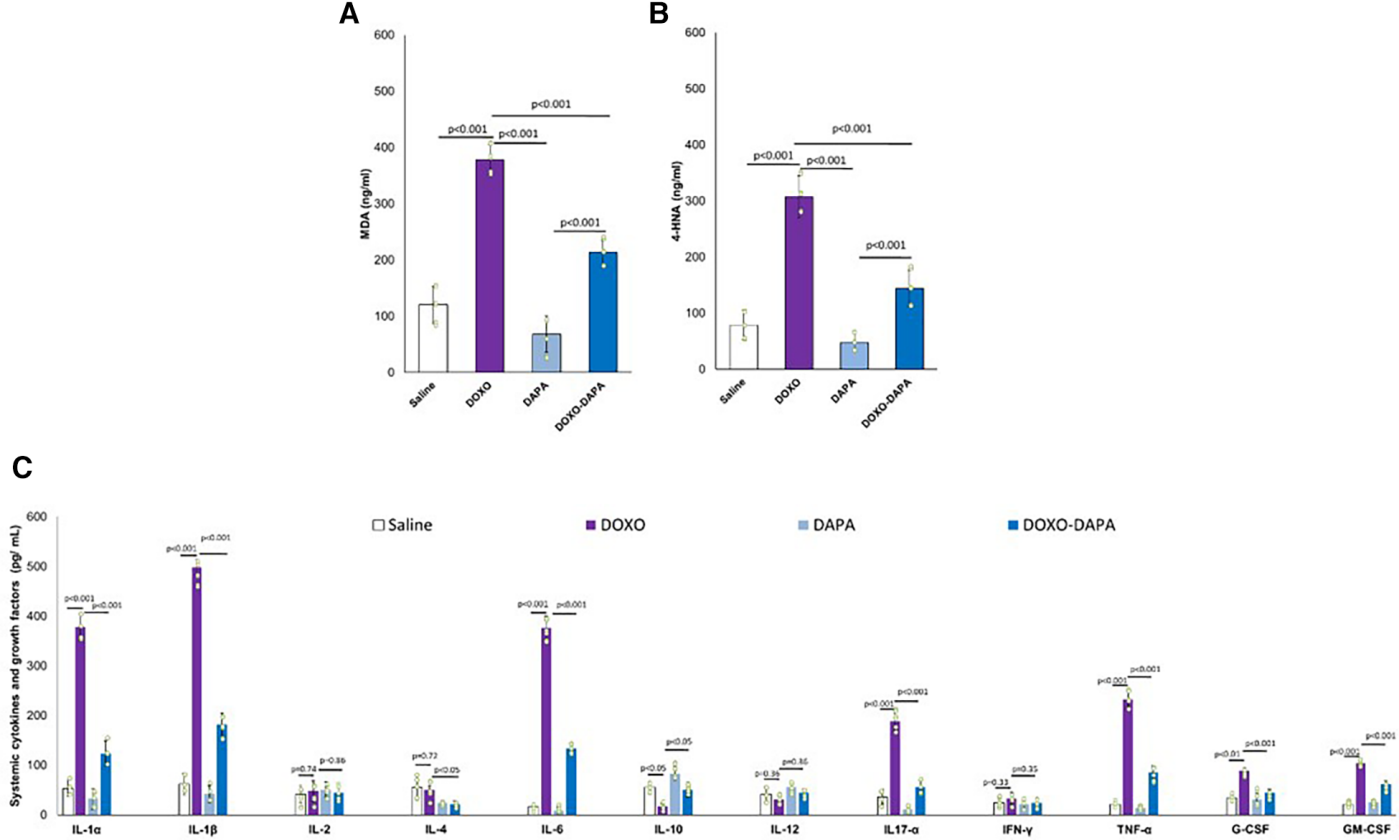
Schematic representation of DAPA-related cytoprotective properties in cardiomyocytes. SGLT2i reduces both glucose and sodium intake leading to intracellular hyponatremia in the cardiomyocyte. Lower Na^+^ levels leads to a reduced function of sodium-calcium exchanger, resulting in low levels of intracellular calcium. Preserving excess intracellular calcium improves the mitochondrial functions of cardiomyocyte, optimizing ATP production. Furthermore, DAPA has antioxidant effects, counteracting lipid peroxidation and the intracellular concentration of iROS, reducing ferroptosis and the consequent release of cardiac troponins. Moreover, DAPA reduces the expression of MyD-88 and NLRP3 in the cardiomyocyte, counteracting the synthesis of pro-inflammatory and cardiotoxic cytokines through NF-kB pathways.

These findings suggest that SGLT2is may have cardioprotective effects in patients treated with anthracyclines. The actual known mechanisms of SGLT2is related to cardioprotective agents involve the reduction of oxidative stress and promotion of sodium and water extraction, leading to reduced cardiac strain (97). From a cellular point of view, in line with the literature, our results on human cardiomyocytes demonstrated that SGLT2i DAPA exerts its cytoprotective and anti-inflammatory properties through the reduction of intracellular Ca^++^ levels, which are able to improve mitochondrial function in cardiomyocytes (98); moreover, DAPA is able to reduce iROS content and lipid peroxidation in cardiac cells, thus preventing ferroptosis. DAPA also exerts anti-inflammatory properties in cardiomyocytes through the reduction of NLRP-3 and Myd-88 pathways, resulting in reduced NF-kB levels and pro-inflammatory cytokines, such as IL-1β, IL-6, and IL-8 (Figure 6) (99). Interestingly, very recent findings indicate potential immune-regulating properties of SGLT2i, such as canagliflozin or empagliflozin; in line with these studies, in activated human peripheral blood mononuclear cells (hPBMC) only, a significant reduction of IL-2 secretion was seen in DAPA-exposed immune cells (Supplementary Figure S1), indicating potential immune effects of SGLT2i. These properties should be more detailed and could be of great interest in finding new immune-modulating agents in autoimmune patients or for the prevention and treatment of myocarditis, vasculitis, and endothelitis induced by viruses or immune checkpoint inhibitors (ICIs) in cancer patients (100).

**Figure 7:**
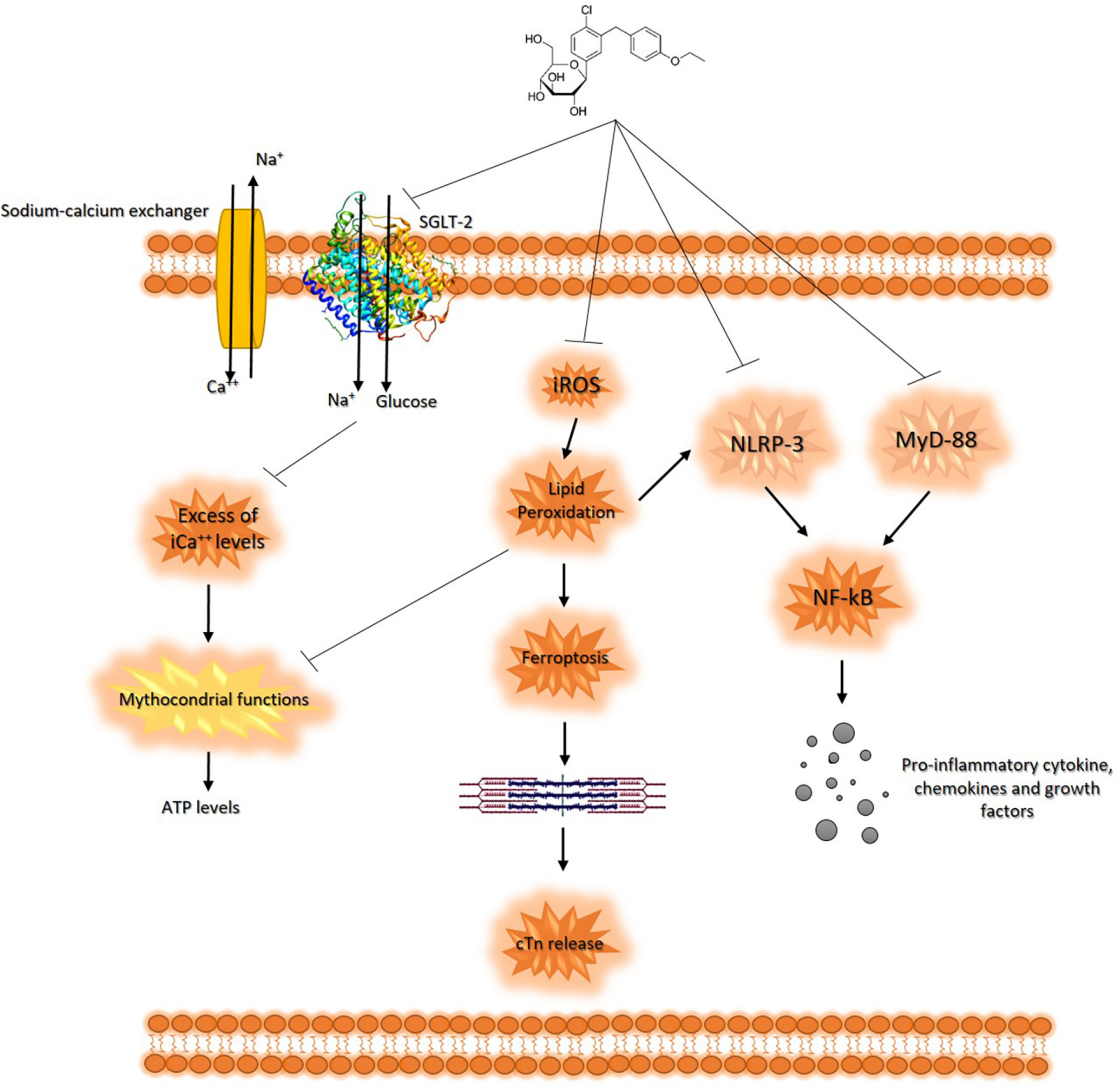
MDA and 4-HNA (**A,B**) systemic levels, and systemic cytokines (**C**) in mice treated with saline solution (control), DOXO 2.17 mg/kg/day, DAPA 10 mg/kg/day, and DOXO-DAPA in association (*n* = 6 for each group). One-way ANOVA. Values are expressed as mean ±SD.

Moreover, in this study, for the first time, the different beneficial effects of DAPA were analyzed in preclinical models of anthracycline-induced cardiotoxicity. In line with other studies, DAPA demonstrated both systemic and cardio-renal anti-inflammatory effects. Recently, Gongora et al. (15) performed a retrospective study to test the preventive properties of cardiac dysfunctions and overall safety of SGLT2i in more than 3,000 cancer patients with T2DM treated with anthracyclines. The primary cardiac outcome was a composite of cardiac events [heart failure incidence, heart failure admissions, new cardiomyopathy (>10% decline in ejection fraction to <53%) and clinically significant arrhythmias]; the primary safety outcome was overall mortality. There were 20 cardiac events over a median follow-up period of 1.5 years. The incidence of cardiac events was lower among case patients in comparison to control participants (3% vs. 20%; *p* = 0.025). Patients treated with SGLT2is patients also experienced lower overall mortality when compared with control participants (9% vs. 43%; *p* < 0.001) and a lower composite of sepsis and neutropenic fever (16% vs. 40%; *p* = 0.013). This study demonstrated, for the first time, the abilities of SGLT2i in the prevention of cardiac dysfunctions in cancer patients with no relevant toxicities (15). Another more recent observational study (96) concluded that dapagliflozin is well-tolerated and associated with high compliance in patients with advanced, inoperable pancreatic ductal adenocarcinoma, significantly reducing some cancer-associated biomarkers (96). Systemic inflammation, also known as systemic inflammatory response syndrome (SIRS), can occur in cancer patients treated with doxorubicin (101). Cancer patients treated with DOXO experienced high levels of CRP, erythrocyte sedimentation rate, IL-6, and IL-1β that may contribute to additional complications, including organ dysfunction or failure (102, 103). DAPA significantly reduced the biomarkers of inflammation and of heart failure, including troponins and NT-pro-BNP, confirming systemic anti-inflammatory and cardioprotective properties. Myocardial analysis showed that DAPA reduced NLRP3 and Myd-88 expression in heart tissue. NLRP3 inflammasome and Myd-88 activation have been implicated in several diseases, including cancer and cardiomyopathies (Figure 7). Both induce cardiomyocyte death and exacerbate myocardial injury by promoting inflammation and fibrosis through IL-1β and IL-18, which activates macrophages and immune cells in heart tissue (104).

**Figure 8:**
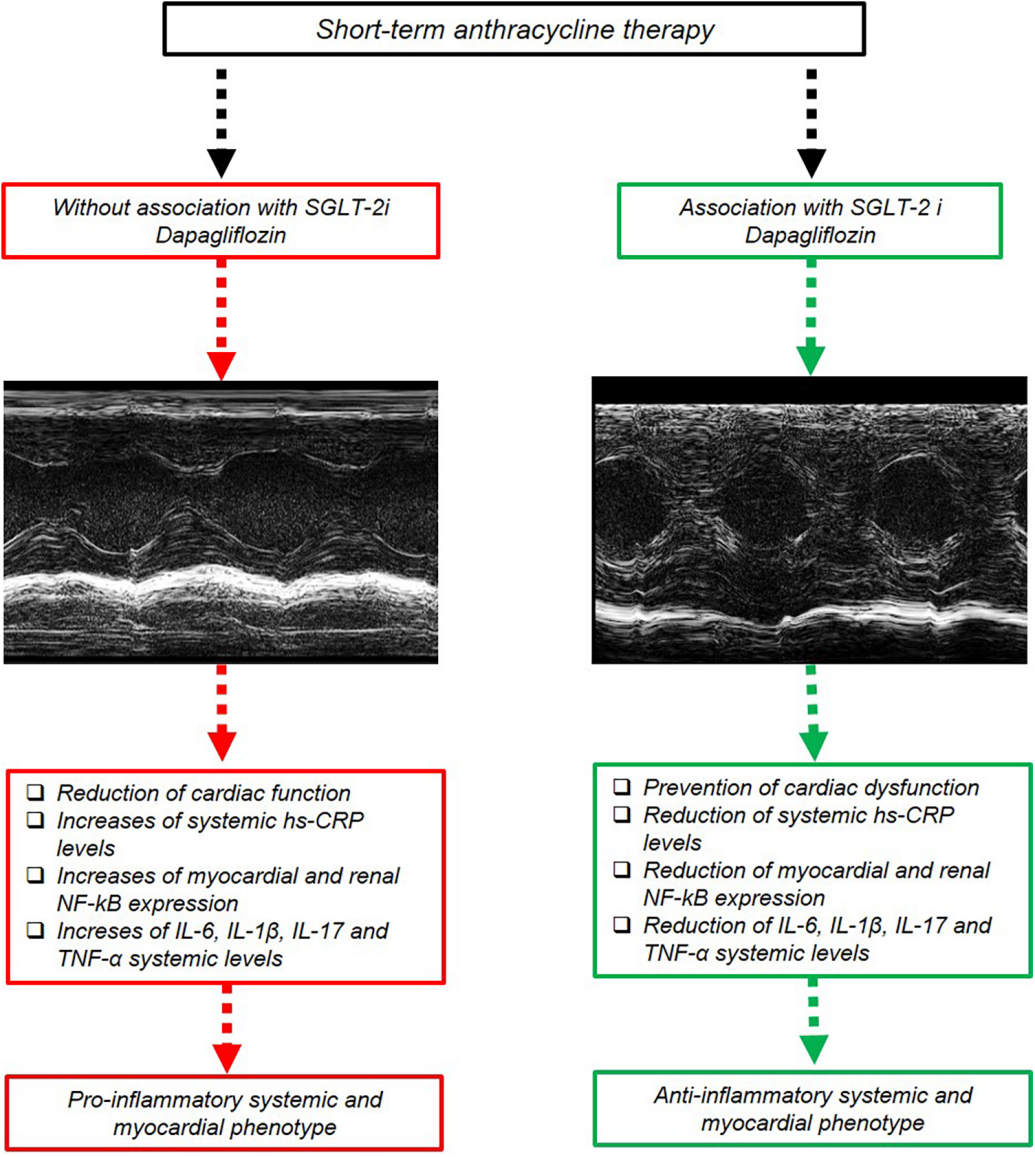
Schematic representation of DAPA-mediated cardioprotective and anti-inflammatory effect in preclinical models. Short-term DOXO therapy reduced systolic cardiac function; increased systemic hs-CRP, IL-6, IL-1β, IL-17, and TNF-α levels; and increased myocardial and kidney NF-kB expression. DAPA attenuated DOXO-induced phenotype through inhibition of NLRP-3 and Myd-88 pathway, resulting in preservation of cardiac function and reduced systemic levels of hs-CRP, IL-6, IL-1β, IL-17, and TNF-α.

Moreover, activation of NF-κB has been implicated in the inflammatory response and development of cardiac injuries (105); DOXO increases myocardial reactive oxygen species that can activate NF-κB signaling. Once activated, NF-κB translocates into the nucleus and promotes the expression of various pro-inflammatory genes, including cytokines, chemokines, and adhesion molecules involved in heart failure and fibrosis (106). Overall, NF-κB activation plays a significant role in doxorubicin-induced cardiotoxicity by mediating the inflammatory response and modulating cell survival pathways (107). To the best of our knowledge, this is the earliest evidence that DAPA is able to suppress NF-Kb expression in myocardial and renal tissue through IHC methods in preclinical models of DOXO cardiotoxicity. The overall picture of the study (Figure 8) summarizes the potential systemic and cardio-renal benefits of DAPA in preclinical models of cardio-oncology.

The present study has some limitations. First, this is a preliminary indication that DAPA could prevent cardiac dysfunctions and decrease biomarkers of cardiotoxicity in preclinical models of short-term-induced cardiomyopathies; however, a detailed mechanistic study of DAPA-related cardioprotection should be carried out, through the use of selective inhibitors of intracellular pathways potentially involved in beneficial properties of DAPA (i.e., through the use of NLRP-3 and MyD-88 selective inhibitors). Second, DOXO-induced cardiotoxicity also occurs many years after chemotherapy (108), especially in young women with breast cancer. Therefore, the long-term effects of DAPA in preclinical models exposed to anthracyclines should be performed; however, acute, short-term, cardiac, and endothelial biochemical changes are frequently seen in these patients and are clinically relevant. On the other hand, we studied the early effects of DAPA on the myocardial metabolism of preclinical models without assessing insulin levels, homeostatic model assessment (HOMA)-index, and ketogenic bodies (SGLT2is increase acetate and butyrate systemic levels that could affect myocardial metabolism) (109). Moreover, this study focalized the cardiovascular benefits only in female preclinical models, in line with other similar studies in cardio-oncology (34, 37–39). Anthracycline-induced cardiotoxicity is frequently seen in female breast cancer patients; therefore, a preclinical female model to mimic the clinical condition that we frequently observe in cardio-oncology was used, i.e., women with breast cancer treated with anthracyclines who develop cardiomyopathies. However, subsequent studies will be performed also in male mouse models to evaluate the impact of sex difference (110) in DAPA cardioprotection.

Currently, there is a need for cardioprotective strategies in cancer patients treated with doxorubicin, considering its relevant cardiotoxicity (111). The cardiovascular benefits (e.g., HHF and cardiovascular death) of SGLT2is are different and the mechanisms are partially elucidated. Recent clinical evidence of SGLT2is in cancer patients with T2DM indicate that gliflozins could reduce cardiovascular mortality, MACE, and hospitalization for heart failure. The data in the present study recommend the use of DAPA in the primary prevention of anthracycline-induced cardiotoxicities in cancer patients without diabetes, consequently reducing the discontinuation of therapies, hospitalizations for cardiovascular diseases, and the index of relevant cardiotoxic events.

The present study highlights the mechanisms of DAPA-mediated cardio-renal benefits in preclinical models of anthracycline toxicity. We provide new insight into the cardiovascular benefits of DAPA, as our data show that DAPA induced an anti-inflammatory systemic phenotype during DOXO therapy, reducing NF-kB expression in myocardial and kidney tissue. The overall picture of the study encourages the use of DAPA in non-diabetic cancer patients treated with anthracyclines to prevent adverse cardiac events. Further studies are warranted to investigate interconnected pathophysiological mechanisms of DAPA-induced cardioprotection.

## Data Availability

The datasets presented in this study can be found in online repositories. The names of the repository/repositories and accession numbers can be found here: https://zenodo.org/record/8119945.
