# Supplementary material for: Sodium-glucose cotransporter 2 inhibitor dapagliflozin prevents ejection fraction reduction, reduces myocardial and renal NF-κB expression and systemic pro-inflammatory biomarkers in models of short-term doxorubicin cardiotoxicity
Source: Front Cardiovasc Med. 2024 May 16;11:1289663. doi: 10.3389/fcvm.2024.1289663 (PMC11138344; doi:10.3389/fcvm.2024.1289663)
Supplement: Supplementary file 1 [file Datasheet1.docx]

**Supplementary file**

**Materials and methods**

**2.1 IL-2 secretion by activated hPBMC exposed to DAPA**

To assess putative immune effects of DAPA, primary peripheral blood mononuclear cells (hPBMCs), a heterogeneous population of blood cells including macrophages, dendritic cells, monocytes and lymphocytes (ATCC #PCS-800-011, USA) were grown in 96-well flat-bottom plates at the density of 150000 cells/well for 16 h. In line with literature (25), cells were cultured in human plasma like medium (HPLM; Gibco), and unstimulated (control) or activated in the presence of anti-CD3 (2 μg/ml; Biolegend) and soluble anti-CD28 (20 μg/ml; Biolegend) and unexposed or exposed to DAPA at 1, 10 and 100 nM for 24h. After treatments, cells were centrifuged at 1500 rpm for 15 min, supernatants were analyzed for quantification of secreted IL-2 through selective ELISA (Thermo Fisher, Milan, Italy,ssay range 0.0128 - 5000 pg/mL).

**Results**

To assess potential effects of DAPA in immune cells, hPBMC were exposed to DAPA for 24h under exposure to anti CD3 and CD28 mAbs, in line with a very recent study on antidiabetic drugs and canagliflozin (see ref in 26,46 in the main manuscript file). IL-2 release in supernatant was different between groups (Figure 1 I); interestingly, secreted IL-2 by hPBMS was significantly reduced only in DAPA 10 and 100 nM groups (1202 ±94.2 and 969.7 ±81.6 pg/ml compared to 16225 ±113.3 pg/ml of only activated hPBMS).


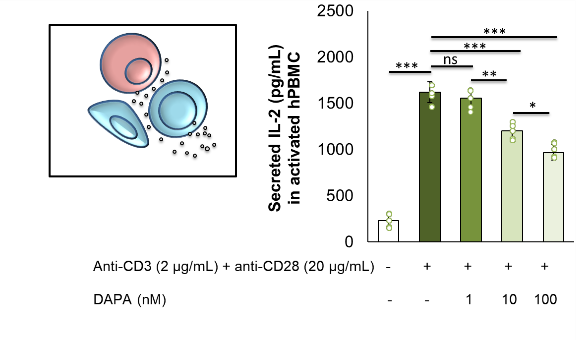


Fig.1 Supplementary: IL-2 secretion by activated ( through anti CD3 + anti CD28 antibodies) or unactivated hPBMC exposed or unexposed to DAPA (1, 10 or 100 nM). One way ANOVA. Values are expressed ± SD. *** P<0.001; **P<0.01; *P<0.05; ns: not significant.
